# Supplementary material for: Urban water systems: Development of micro-level indicators to support integrated policy
Source: PLoS One. 2020 Feb 24;15(2):e0228295. doi: 10.1371/journal.pone.0228295 (PMC7039670; doi:10.1371/journal.pone.0228295)
Supplement: S1 File — (DOCX) [file pone.0228295.s001.docx]

**Urban water systems: Supplementary information**

Table 1: DKI Jakarta urban water security indicators data details and sources

| Code | Indicator | Metric | Range/  Scale/  Value | Unit | Legend | Year | Coverage -number of kelurahan | Source | Access | Data transformation/derivation |
| --- | --- | --- | --- | --- | --- | --- | --- | --- | --- | --- |
| 1000 | **PRESSURE INDEX** | **-** | **-** | **-** | **-** | **-** | **-** | **-** | **-** | **-** |
| 1100 | **Environmental pressures** | **-** | **-** | **-** | **-** | **-** | **-** | **-** | **-** | **-** |
| 1101 | Surface water availability | Reservoir volume,  city-level | 234,160 | m^3^ | - | - | - | [1] | - | - |
| 1102 | Precipitation (annual) | City-level | 1816 | mm | - | - | - | - | - | - |
| 1103 | Rainfall variability | City-level | ±43 – 300 | mm | - | - | - | - | - | - |
| 1104 | Land surface hazard (storm surge, riverine flood) | Elevation | -5 to 44 | Metres | NA | 2008 | 260 | [2] | Open-sourced | The minimum elevation of the kelurahan was chosen to represent the elevation of the kelurahan. Elevation data also represents vulnerability to hazards such as storm surges and floods. |
| 1200 | **Socioeconomic pressures** | **-** | **-** | **-** | **-** | **-** | **-** | **-** | **-** | **-** |
| 1201 | Population growth | Population growth | 0 to 100 | % | NA | 2013-2014 | 260 | [3] | Open-sourced | Population data from 2013 and 2014 were used. |
| 1202 | Slums | Slum density | 0 to 100 | % | NA | 2013 | 260 | [4] | Open-sourced | The number of slum households is expressed as a percentage of the total number of households in the kelurahan. |
| 1203 | Economic activity | Night-time light (NTL) radiance | 10 to 120 | Radiance | NA | 2016 | 260 | [5] | Open-sourced | The NTL images are captured by the Day/Night Band (DNB) in the Visible Infrared Imaging Radiometer Suite (VIIRS) satellite launched in 2011. The 2016 cloud-free, outlier-removed, average radiance composite was used, with background (non-lights) set to zero (“vcm-orm-ntl” product). This product has been found to best represent the brightness of human development [6]. Stray light, lightning, lunar illumination and cloud cover were filtered out before obtaining the average radiance. Radiance values from the monthly composites in the year are averaged to derive the annual composite.  To obtain the average radiance values of the kelurahan in Jakarta, the Zonal Statistics as Table tool in ArcGIS was used with the inputs being the NTL dataset and Jakarta kelurahan boundaries purchased from the BPS. A pixel in considered to be within a kelurahan if it’s centrepoint falls within the kelurahan boundary. Average radiance of the kelurahan is the average radiance of the pixels within the kelurahan. |
| 1204 | Non-domestic demand | Water usage of small-medium industries | 0 to 2 | Scale | 0= No small-medium industries 1= low/moderate water usage 2= high water usage | 2014 | 260 | [7] | Open-sourced | Small-medium industries are geolocated and assigned a water intensity level (low to high) based on the product output of the industry. |
| 1205 | Industrial activity | Industrial zones | Binary (0/1) | Yes/No | 0= No industrial zone 1= Presence of industrial zone | 2010-2030 | 260 | [8] | Open-sourced | Kelurahan are categorised as industrial if more than 50% of the area of the kelurahan falls in an industrial zone. |
| 2000 | **STATE INDEX** | **-** | **-** | **-** | **-** | **-** | **-** | **-** | **-** | **-** |
| 2100 | **Water Service** | **-** | **-** | **-** | **-** | **-** | **-** | **-** | **-** | **-** |
| 2101 | Piped water access | Piped water network coverage | 0 to 100 | % | NA | 2017 | 260 | PAMJAYA | Restricted | To derive the extent of piped water network coverage, the number of customers in a kelurahan is multiplied by 5 to represent the population served by piped water in the kelurahan. This figure is expressed as a percentage of the total kelurahan population. |
| 2102 | Piped water pressure | Piped water pressure (% time low pressure) | 5 categories | % | NA | 2016-2017 | 259 | [9,10] | Purchased | The piped water pressure was derived from several maps by BRPAMDKI (DKI Jakarta Water Supply Services Regulatory Body) showing piped water consumption according to zones established by the water service providers or PAMJAYA. Maps are available for 4 months: January, September and December 2016 and March 2017. These four maps are georeferenced to allow the piped water pressure data to be mapped to each kelurahan. For some months, there are some blank areas with no pressure data. As such, a combination of all four of these maps are used to obtain maximum coverage of pressure data. Only data for 1 kelurahan is missing from combining these four maps. The pressure data for each kelurahan is represented by the number of months piped water pressure is less than 0.75 atm, expressed as a percentage of the total number of months that pressure data is available for that kelurahan. |
| 2103 | Affordability | City-level | 4 | % of average monthly income | - | - | - | [1] | - | - |
| 2200 | **Water Quality** | **-** | **-** | **-** | **-** | **-** | **-** | **-** | **-** | **-** |
| 2201 | Drinking water quality | City-level | 97.5 | % meet standards | - | - | - | [10] | - | - |
| 2202 | Groundwater quality | Groundwater conservation zone classification | 1 to 3 | Scale | 1= GW Recharge/Safe 2= Prone 3= Critical/Damaged | 2017 | 260 | [11] | Open-sourced | The GW Conservation Zones 2017 were developed by the Groundwater Conservation Agency (BKAT) in Jakarta, where it captures groundwater quality and quantity (Table 2). Where a kelurahan comprises of 2 or more GW zones, the zone which occupies the largest area in the kelurahan is chosen to represent its GW condition. |
| 2300 | **Sanitation infrastructure** | **-** | **-** | **-** | **-** | **-** | **-** | **-** | **-** | **-** |
| 2301 | Wastewater disposal | Population with access to own septic tank | 0 to 100 | % | NA | 2010 | 260 | Indonesia Census, BPS | Purchased | No manipulation of data. |
| 2400 | **Flood protection infrastructure** | **City-level (qualitative)** |  |  |  |  |  |  |  |  |
| 3000 | **IMPACT INDEX** | **-** | **-** | **-** | **-** | **-** | **-** | **-** | **-** | **-** |
| 3100 | **Water Supply** | **-** | **-** | **-** | **-** | **-** | **-** | **-** | **-** | **-** |
| 3101 | Access to safe water | Population using protected drinking water sources | 0 to 100 | % | NA | 2010 | 260 | Indonesia Census, BPS | Purchased | Protected water sources consist of piped, bottled, and pumped water as the main drinking water source. |
| 3102 | Reliance on groundwater | Groundwater consumption | 0 to 10 | lcd | NA | 2018 | By kecamatan per capita | [12] | Open-sourced | Groundwater consumption data was only available at the kecamatan level. The latest available annual groundwater consumption data by kecamatan was divided by the number of days in 2018 (365) and the kecamatan population to obtain daily groundwater consumption per capita (lcd) in that kecamatan. |
| 3200 | **Health** | **-** | **-** | **-** | **-** | **-** | **-** | **-** | **-** | **-** |
| 3201 | Sanitation access | Population with access to toilet | 0 to 100 | % | NA | 2010 | 260 | Indonesia Census, BPS | Purchased | No manipulation of data. |
| 3202 | Waterborne disease risk | Diarhoea prevalence rate | 0 to 950 | No. of cases per 10,000 people | NA | 2018 | 260 | [13] | Open-sourced | The number of diarhoea cases in 2018 is expressed as prevalence rates using the latest available population data in 2017. |
| 3203 | Water-related disease risk | Dengue prevalence rate | 0 to 17 | No. of cases per 10,000 people | NA | 2018 | 260 | [13] | Open-sourced | The number of dengue cases in 2018 is expressed as prevalence rates using the latest available population data in 2017. |
| 3300 | **Environment** | **-** | **-** | **-** | **-** | **-** | **-** | **-** | **-** | **-** |
| 3301 | Groundwater over-exploitation | Change in Groundwater Conservation Zone | -1 to 1 | Scale | -1= Worsened 0= Unchanged 1= Improved | 2013-2017 | 260 | [11] | Open-sourced | A comparison is done for each kelurahan between its Groundwater Conservation Zone in 2013 and 2017 to reflect any improvement or worsening of groundwater quality and/or quantity in that kelurahan. |
| 3302 | Flood incidence | Number of years flooded between 2013 to 2016 | 0 to 4 | Scale | Numbers indicate number of years | 2013-2016 | 260 | [14–17] | Open-sourced | No manipulation of data. |
| 4000 | **RESPONSE INDEX** | **-** | **-** | **-** | **-** | **-** | **-** | **-** | **-** | **-** |
| 4001 | Governance framework | City-level (qualitative) | - | - | - | - | - | - | - | - |
| 4002 | Policy framework | City-level (qualitative) | - | - | - | - | - | - | - | - |

Table 2: Groundwater Conservation Zone Classification [11]

| **Groundwater Conservation Zone** | **% vol of water** | **TDS (mg/L)** | **Electrical conductivity** | **Heavy metals** |
| --- | --- | --- | --- | --- |
| **Safe** | <40 | <1000 | <1000 | 0 |
| **Prone** | 40-60 | 1000-10,000 | 1000-1500 | 0 |
| **Critical** | 60-80 | 10,000-100,000 | 1500-5000 | 0 |
| **Damaged** | >80 | >100,000 | >5000 | Present |

**References**

[1] BPPSPAM (Badan Peningkatan Penyelenggaraan Sistem Penyediaan Air Minum). Kinerja PDAM 2018: Wilayah II 2018.

[2] DEMNAS (DEM Nasional). Seamless Digital Elevation Model (DEM) dan Batimetri Nasional 2008. http://tides.big.go.id/DEMNAS/ (accessed July 12, 2019).

[3] Portal Data Terpadu Pemprov DKI Jakarta. Data Jumlah Penduduk Berdasarkan Kewarganegaraan dan Jenis Kelamin Per Kelurahan - data.jakarta.go.id. Jkt Open Data 2013. http://data.jakarta.go.id/dataset/jumlahpendudukberdasarkanjeniskelamindankewarganegaraandkijakarta (accessed July 12, 2019).

[4] BPS (Badan Pusat Statistik). Evaluasi Rukun Warga (RW) Kumuh Provinsi DKI Jakarta 2013 2013.

[5] NCEI (National Centers for Environmental Information), NOAA (National Oceanic and Atmospheric Administration). Version 1 VIIRS Day/Night Band Nighttime Lights n.d. https://www.ngdc.noaa.gov/eog/viirs/download_dnb_composites.html (accessed July 12, 2019).

[6] Elvidge CD, Baugh K, Zhizhin M, Hsu FC, Ghosh T. VIIRS night-time lights. Int J Remote Sens 2017;38:5860–79. doi:10.1080/01431161.2017.1342050.

[7] Portal Data Terpadu Pemprov DKI Jakarta. Data Daftar Sentra Industri Kecil Dan Menengah DKI Jakarta. Jkt Open Data 2014. http://data.jakarta.go.id/dataset/daftar-sentra-industri-kecil-dan-menengah (accessed July 12, 2019).

[8] BAPPENAS (Badan Perencanaan Pembangunan Nasional). RTRW Kota/Kab. Administrasi Provinsi DKI Jakarta 2030 2015.

[9] BRPAMDKI (Badan Regulator Pelayanan Air Minum DKI Jakarta). Kinerja Kuartal III/2016 : Tekanan dan Kualitas Air Minum Jakarta 2016. http://www.brpamdki.org/peformance-2016/detail/105/ (accessed July 12, 2019).

[10] BRPAMDKI (Badan Regulator Pelayanan Air Minum DKI Jakarta). Kinerja Kuartal I /2017 : Tekanan dan Kualitas Air Minum Jakarta 2017. http://www.brpamdki.org/peformance-2017/detail/190/ (accessed July 12, 2019).

[11] BKAT (Balai Konservasi Air Tanah). Pengelolaan Air Tanah Di Cekungan Air Tanah Jakarta: Permasalahan dan Kemajuan Penyelesaian Masalah n.d.

[12] Portal Data Terpadu Pemprov DKI Jakarta. Data Penggunaan Air Tanah pada Pelanggan Air Tanah di DKI Jakarta. Jkt Open Data 2018. http://data.jakarta.go.id/dataset/data-penggunaan-air-tanah-pada-pelanggan-air-tanah-di-dki-jakarta (accessed July 12, 2019).

[13] Dinas Kesehatan DKI Jakarta. Data tabular dari PWS KLB (W2) Kelurahan. Seksi Survailans Epidemiol 2018. http://surveilans-dinkesdki.net/ (accessed July 12, 2019).

[14] Portal Data Terpadu Pemprov DKI Jakarta. Data Kejadian Bencana Banjir Tahun 2013 di DKI Jakarta. Jkt Open Data 2013. http://data.jakarta.go.id/dataset/rekap-banjir-bulan-februari-2016 (accessed July 12, 2019).

[15] Portal Data Terpadu Pemprov DKI Jakarta. Data Kejadian Bencana Banjir Tahun 2014 di DKI Jakarta. Jkt Open Data 2014. http://data.jakarta.go.id/dataset/rekap-banjir-bulan-januari-2016 (accessed July 12, 2019).

[16] Portal Data Terpadu Pemprov DKI Jakarta. Data Kejadian Bencana Banjir Tahun 2015 di DKI Jakarta. Jkt Open Data 2015. http://data.jakarta.go.id/dataset/data-rekapitulasi-kejadian-banjir-bulan-januari-2015 (accessed July 12, 2019).

[17] Portal Data Terpadu Pemprov DKI Jakarta. Data Kejadian Bencana Banjir Tahun 2016 di DKI Jakarta. Jkt Open Data 2016. http://data.jakarta.go.id/dataset/rekap-banjir-tahun-2016 (accessed July 12, 2019).
