# Supplementary material for: Urban water systems: Development of micro-level indicators to support integrated policy
Source: PLoS One. 2020 Feb 24;15(2):e0228295. doi: 10.1371/journal.pone.0228295 (PMC7039670; doi:10.1371/journal.pone.0228295)
Supplement: S2 File — (DOCX) [file pone.0228295.s002.docx]

**Urban water systems: development of micro-level indicators to support integrated policy**

Olivia Jensen and Adilah Khalis

Note: Development of Radiance Indicator

Night-time lights (NTL) data is an open-sourced geodataset from the United States Earth Observation Group (EOG), National Centers for Environmental Information (NCEI), National Oceanic and Atmospheric Administration (NOAA). The NTL images are captured by the Day/Night Band (DNB) in the Visible Infrared Imaging Radiometer Suite (VIIRS) satellite launched in 2011. For this paper, the 2016 cloud-free, outlier-removed, average radiance composite was used, with background (non-lights) set to zero (“vcm-orm-ntl” product). This product has been found to best represent the brightness of human development (Elvidge et al. 2017). Stray light, lightning, lunar illumination and cloud cover were filtered out before obtaining the average radiance. Radiance values from the monthly composites in the year are averaged to derive the annual composite.

To obtain the average radiance values of all villages/kelurahan in Jabodetabek, the Zonal Statistics as Table tool in ArcGIS was used with the inputs being the NTL dataset and Jabodetabek villages/kelurahan boundaries obtained from the Indonesian national statistical body (BPS). A pixel in considered to be within a village/kelurahan if its centrepoint falls within the village/kelurahan boundary. Average radiance of the village/kelurahan is the average radiance of the pixels within the village/kelurahan.

*Check out: (Ma, Zhou, Pei, Haynie, & Fan, 2012; Zhang & Seto, 2011) from Mertes et al. 2015*

**References**

VIIRS DNB download link: <https://www.ngdc.noaa.gov/eog/viirs/download_dnb_composites.html>

Elvidge, Christopher D, Kimberly Baugh, Mikhail Zhizhin, Feng Chi Hsu, and Tilottama Ghosh. 2017. ‘VIIRS Night-Time Lights’. *International Journal of Remote Sensing* 38 (21): 5860–79. https://doi.org/10.1080/01431161.2017.1342050.

Mertes, C.M., A. Schneider, D. Sulla-Menashe, A.J. Tatem, and B. Tan. 2015. ‘Detecting Change in Urban Areas at Continental Scales with MODIS Data’. *Remote Sensing of Environment* 158 (March): 331–47. https://doi.org/10.1016/j.rse.2014.09.023.
